# Genomic Selection in Winter Wheat Breeding Using a Recommender Approach

**DOI:** 10.3390/genes11070779

**Published:** 2020-07-11

**Authors:** Dennis N. Lozada, Arron H. Carter

**Affiliations:** 1 Department of Crop and Soil Sciences, Washington State University, Pullman, WA 99164, USA; den.lozada@wsu.edu or; 2 Department of Plant and Environmental Sciences, New Mexico State University, Las Cruces, NM 88003, USA

**Keywords:** Bayesian models, genomic BLUP (GBLUP), grain yield, heading date, high-throughput phenotyping, item-based collaborative filtering (IBCF), plant height, recommender system, snow mold tolerance, spectral reflectance indices

## Abstract

Achieving optimal predictive ability is key to increasing the relevance of implementing genomic selection (GS) approaches in plant breeding programs. The potential of an item-based collaborative filtering (IBCF) recommender system in the context of multi-trait, multi-environment GS has been explored. Different GS scenarios for IBCF were evaluated for a diverse population of winter wheat lines adapted to the Pacific Northwest region of the US. Predictions across years through cross-validations resulted in improved predictive ability when there is a high correlation between environments. Using multiple spectral traits collected from high-throughput phenotyping resulted in better GS accuracies for grain yield (GY) compared to using only single traits for predictions. Trait adjustments through various Bayesian regression models using genomic information from SNP markers was the most effective in achieving improved accuracies for GY, heading date, and plant height among the GS scenarios evaluated. Bayesian LASSO had the highest predictive ability compared to other models for phenotypic trait adjustments. IBCF gave competitive accuracies compared to a genomic best linear unbiased predictor (GBLUP) model for predicting different traits. Overall, an IBCF approach could be used as an alternative to traditional prediction models for important target traits in wheat breeding programs.

## 1. Introduction

Genomic selection (GS) uses genome-wide marker information to predict the genomic estimated breeding values (GEBV) of individuals [1]. In GS, a population with known phenotype and genotype information (training population) is used to predict the GEBV of individuals having only genotype data (test or validation population) [2]. GEBV can be then used as a basis for selecting which lines to advance and/or be used as parents in the breeding program. The utility of GEBV for choosing parents for the next generation of crossing, together with the introgression of favorable alleles, can be used to fast-forward genetic gain [3]. The performance of GS depends on predictive ability, defined as the correlation between GEBV and observed phenotype [4,5]. The predictive power of GS is a factor of genetic relatedness between the training and test populations, prediction models used, number of markers, population structure, including secondary correlated traits in the model, among others [6,7,8,9,10].

In recent years, recommender algorithms have been developed to streamline the selection process in electronic commerce (e-commerce) based on the customer’s preferences through generating a list of recommended items [11,12]. Item-based collaborative filtering (IBCF) is a popular recommender approach for suggesting which items to buy [13]. In this process, relationships between the different items are first identified, and this information is subsequently used to compute recommendations, where a database of preferences for users is ultimately established [11]. In an IBCF recommender system, a set of items that has been rated (i.e., selected or bought) by a specific user *u_i_* is first evaluated and compared to the target item *Y*; after which, the most similar items to *Y* are selected. The similarity coefficients between the most similar items are then computed, and once the most similar items are identified, prediction for the *u_i_* and the target item *Y* is calculated through a simple weighted average of the similar items [13]. The IBCF recommender system has been recently used in the context of genomic selection (GS) to predict performance of wheat lines in multi-trait, multi-environment trials [13,14,15]. In an IBCF-GS approach, phenotypic data for each line *i* for every trait–environment combination *j* is first built. Afterward, raw phenotypic data from each line is standardized using the mean and standard deviation for each *j*. Using the correlation (similarity) coefficients between each *j*, scaled predictive phenotype for each *i* on a trait–environment combination, and the adjusted trait values, predicted values for the missing phenotypes are calculated [16].

A key to the successful evaluation and implementation of GS is an improved predictive ability for target traits [13]. Hence, to make GS more relevant in the breeding program, it is necessary to explore alternative models which could potentially result in improved predictive ability. As more empirical evidence is required to get a clearer picture of the performance of the IBCF recommender approach [16], it would be essential to assess how this system would be useful in the context of GS in a winter wheat breeding program. Therefore, the objectives of this study were to (1) evaluate the potential of using an IBCF recommender system for GS of different traits in winter wheat; (2) predict grain yield using secondary spectral traits from high-throughput phenotyping using an IBCF approach; and (3) compare the predictive ability of IBCF with a univariate genomic BLUP (GBLUP) prediction model.

## 2. Materials and Methods

### 2.1. Experimental Datasets

An association mapping panel consisting of 456 winter wheat lines adapted to the Pacific Northwest region of the US was evaluated for different grain yield, agronomic, spectral reflectance, and disease resistance traits between the 2015 and 2019 growing seasons across various locations in the state of Washington, US, namely, Lind (LND), Pullman (PUL), Waterville (WAT), and Mansfield (MAN), as described previously [17,18,19]. Grain yield (GY), plant height (PH), and heading date (HD) data collection and analyses for an augmented design were previously described in Lozada and Carter [20]. Briefly, grain yield (in t ha^−1^) was collected by harvesting whole plots using a Zurn 150 Plot combine (Waldenburg, Germany), whereas plant height (in cm) was reported as the measurement from the ground to the tip of the spike, excluding the awn when present. Heading date was recorded as the date when 50% of the plants on each plot had fully visible spikes, reported in Julian days. Best linear unbiased estimates (BLUE) were calculated for single environments, whereas best linear unbiased predictors (BLUP) were calculated for combined analyses across environments under an augmented complete block design using the ACBD [21] software in R [22]. Spectral reflectance indices, namely, Normalized Difference Vegetative Index (NDVI), Normalized Water Index-1 (NWI-1), and Simple Ratio (SR) were calculated from different wavelengths of light (between 430 and 970 nm), collected using a CROPSCAN multispectral radiometer (Rochester, MN, USA) across different developmental stages, namely, heading (Feekes stage 10.2), early grain filling (milking stage; Feekes 10.7), and late grain filling (soft dough stage; Feekes 10.9), as previously described by Lozada et al. [19]. BLUP trait values for the spectral indices across these stages were used to predict grain yield.

Snow mold tolerance (SMT) scores were evaluated in WAT and MAN, based on rating each entry for the disease using a 0–9 scale, where 0 is completely dead, with no green plant tissue visible, and 9 is thriving with no snow mold visible [18]. For these trials, the association mapping panel was planted in paired rows, so that each plot contained two lines—one in the right-side two rows, and one in the left-side two rows [18]. BLUP values for each entry were calculated by combining SMT scores for all environments (ALL) and across years (BLUP17 and BLUP18). BLUE values were derived for each individual environment (MAN17, MAN18, WAT17, and WAT18). Correlation between traits and between environments (*r*) was calculated using the formula *r* = Cov_xy_/√(σ_G_^2^_x_ * σ_G_^2^_y_), where Cov_xy_ is the covariance between two traits (or environments) *x* and *y*; and σ_G_^2^_x_ and σ_G_^2^_y_ are the genotypic variances of traits (or environments) *x* and *y*, respectively [23].

SNP genotyping for the mapping panel was conducted using the Illumina^®^ 90K platform [24] through the USDA-ARS Small Grains Genotyping Laboratory in Fargo, ND, US. After filtering for minor allele frequency (MAF) >0.05 and 20% missing data, 15,229 markers were retained for GS. Out of these number, 12,681 markers (83%) were with known map positions. Marker positions in centimorgans were based on the consensus map reported by Wang et al. [24]. Genotype calls were recorded as 0, 1, 2, where 0 is homozygous for the minor allele, 1 is heterozygous, and 2 is homozygous for the major allele.

### 2.2. Genomic Selection Using a Recommender System

Genomic selection was implemented using the multi-trait, multi-environment item-based collaborative filtering (‘IBCF.MTME’) package developed by Montesinos-López et al. [16] in R software. To illustrate how an IBCF algorithm predicts missing values, suppose we have phenotypic information for multiple lines *i* (1, 2, …, 5) and trait–environment combination *j* (A, B, C, and D) with four missing values denoted by ‘x_(A, B, C, D)n_’ (Table 1). From Table 1, standardized information for each phenotype was derived using the formula [11,14,16]

*Z_ij_* = [(*y_ij_* − *µ_j_*)]/*σ_j_* (1)

to form Table 2. *Z_ij_* is the standardized value for line *i* at trait–environment combination *j*; *y_ij_* is the trait or phenotype value for line *i* at trait–environment combination *j; µ_j_* and *σ_j_* are the mean and standard deviation of the trait–environment combination *j*, respectively. Table 3 presents the correlation (i.e., item-to-item similarity coefficient matrix) between each trait–environment combination.

Using information from Table 1, Table 2 and Table 3, we calculate predicted values for each missing value ‘x’ using the formula:

(2) $${\hat{y}}_{iJ}=\mu_{J}+\sigma_{J}{\hat{z}}_{iJ}$$

where

(3) $${\hat{z}}_{ij}=\frac{\Sigma_{j^{\prime }\in N_{i\left(j\right)}}z_{ij^{\prime }}\;w_{jj^{\prime }}\;}{\Sigma_{j^{\prime }\in N_{i\left(j\right)}}\left|w_{jj^{\prime }}\right|}$$

is the scaled predictive phenotype value for line *i* on trait–environment combination *j*; *N_i_*_(*j*)_ is the item rated as being most similar to trait–environment combination *j*; *w_jj_*_′_ is the weight between trait–environment combinations *j* and *j*′ [16]. From Equations (2) and (3), we calculate values for ‘x_A1_’, ‘x_B2_’, ‘x_C3_’, and ‘x_D4_’ as follows:

*ŷ_xA_*_1_ = 1.63 + 0.28 (1.22) = 1.97

*ŷ_xB_*_2_ = 2.50 + 0.40 (0.90) = 2.86

*ŷ_xC_*_3_ = 1.94 + 0.27 (−0.08) = 1.92

*ŷ_xD_*_4_ = 2.75 + 0.31 (0.07) = 2.77

The IBCF.MTME package implements a random cross-validation, where individuals can appear in both training and testing populations, i.e., these groups are not mutually exclusive [16]. Predictions were performed in four different scenarios following examples presented in Montesinos-López et al. [16]: (1) predictions of performance for the next year or growing season; (2) predictions using multiple environments and traits; (3) predictions using secondary correlated traits; and (4) predictions of breeding values using genomic information from SNP marker data. Predictive ability was represented as the correlation between predicted values and observed phenotypes. Predictive ability from IBCF was compared to predictions using a genomic best linear unbiased prediction (GBLUP) model using Intelligent Prediction and Association tool (iPat) software [25] under 10-fold cross-validations and 10 iterations. GBLUP uses a genomic relationship matrix instead of a pedigree-derived relationship matrix A [26], and is represented by the model *y* = *1µ + Zγ + e*, where *y* is a vector of corrected phenotypic trait values, *µ* is the overall mean, *Z* is an incidence matrix for individual effects, *γ* is vector of breeding values regarded to be in multivariate normal distribution, and *e* is the vector of residual error [27]. Sample files and codes used for performing IBCF analysis in R can be found at https://github.com/dnblozada/IBCF.MTME.

#### 2.2.1. Predicting Performance for the Next Growing Season

In this scenario, GY, HD, and PH on a single year were predicted using information from previous year(s) (refer to Examples 5 and 6 in Montesinos-López et al. [16]). The 2015 growing season was first used as a training dataset to predict the 2016–2019 seasons. Prediction models were then re-trained by adding multiple years to predict performance for the subsequent years in LND and PUL (i.e., 2015_2016 to predict 2017–2019; 2015_2016_2017 to predict 2018 and 2019; and 2015_2016_2017_2018 to predict the 2019 growing season). This scenario simulated a situation where only the information from previous years was available and we wanted to predict information of a trait for a single year using information on the remaining traits across different years [14].

#### 2.2.2. Predictions across Different Environments

Predictions across multiple environments for GY, HD, and PH were conducted by generating 10 random partitions, where 25% of the data was included in the test or validation population whereas the remaining 75% was assigned to the training dataset (see Example 3 in Montesinos-López et al. [16]). Partitions were first generated for cross-validations and then adjustments for phenotypic traits were carried out using the IBCF function.

#### 2.2.3. Predictions Using Secondary Correlated Traits

Grain yield was predicted using secondary correlated traits collected from high-throughput field phenotyping, namely Normalized Difference Vegetative Index (NDVI), Normalized Water Index-1 (NWI-1), and Simple Ratio (SR) using the IBCF algorithm (see Example 4 in Montesinos-López et al. [16]). In this scenario, the dataset was divided into 10 random partitions for cross-validations, where 75% of the data was assigned to the training dataset and the remaining 25% was assigned to the validation or test population. GS for GY was carried out using single and multiple spectral reflectance indices as predictor traits.

#### 2.2.4. Predictions of Breeding Values Using Genomic Marker Information

By default, the IBCF algorithm does not include marker or genomic information to predict missing values. A univariate approach (i.e., trait value for each location was analyzed independently) for phenotypic trait adjustments and predictions of breeding values using Bayesian models in the context of IBCF was implemented [16]. To incorporate marker information in the IBCF.MTME model, two steps were performed. In the first step, phenotypic data for GY, HD, PH, and SMT was adjusted for the markers using simple regression models (Trait ~ *Markers + Error*) implemented in the Bayesian Generalized Linear Regression (BGLR) function [28] in R with 20,000 iterations and 15,000 burn-ins to obtain the breeding values (see Example 8 in Montesinos-López et al. [16]). In the next step, the breeding values of the lines in the validation population were predicted. Cross-validations were conducted for 10 random partitions, where 80% of the lines were used in the training population and the remaining 20% in the test or validation population. Different prediction models were used for trait adjustments, including Bayesian Ridge Regression (BRR), Bayes A, Bayes B, Bayes C, and Bayesian LASSO (Least Absolute Shrinkage and Selection Operator). Features of the different Bayesian regression models are presented in Desta and Ortiz [4] and Wang et al. [26]. The time to execute IBCF algorithm for trait adjustments and breeding value predictions was recorded using the ‘proc.time’ function in R. The predictive ability of this univariate approach for trait adjustments in IBCF was later compared with the predictive power of a univariate GBLUP prediction model.

## 3. Results

### 3.1. Predictive Ability across Different Growing Seasons

Predictions for GY, HD, and PH across different growing seasons using information from previous years resulted in negative to low predictive ability (Table 4). Predictive ability for GY by training different growing seasons to predict subsequent years ranged between −0.21 (PUL 2016_2017 predicting PUL 2018) and 0.46 (LND 2015_2017_2018 predicting PUL 2019), with an average value of 0.08. Mean predictive ability for LND and PUL were 0.16 and 0.03, respectively. Across all environments, predicting the 2019 growing season resulted in the highest mean predictive ability (0.37), followed by 2017 (−0.08), and 2018 (−0.19). Predicting the 2016 growing season in PUL resulted in a mean predictive ability of −0.20.

For HD, predictions resulted in a mean predictive ability of −0.03. Predicting HD for 2018 and 2019 using the 2015–2017 growing seasons resulted in an average predictive ability of 0.07. Predicting the 2016 growing season resulted in a predictive ability of 0.05, whereas predicting 2017, 2018, and 2019 resulted in prediction ability of −0.11, −0.04, and 4.0 × 10^−3^, respectively. Predicting PH resulted in a mean predictive ability of 0.10. Predictive ability ranged between −0.23 (PUL 2015 predicting 2016) and 0.41 (LND 2015_2017_2018 predicting 2019) (Table S1). Similarly, with GY, predicting 2019 across all LND and PUL environments resulted in the highest average predictive ability (0.35). Predicting the other growing seasons (2016–2018) for PH led to low to negative GS predictive ability (between −0.23 and 0.0).

Average correlation values between site-years in LND were 0.21, 0.12, and 0.16 for GY, HD, and PH, respectively (Tables S2–S4). Within PUL, mean correlation was 0.08 (GY), and 0.31 (HD and PH). Mean correlation when 2019 was predicted using the previous growing seasons (2015–2018) was highest for HD (0.36), followed by PH (0.35), and GY (0.14).

### 3.2. Predictive Ability among Different Environments

**Figure 1:**
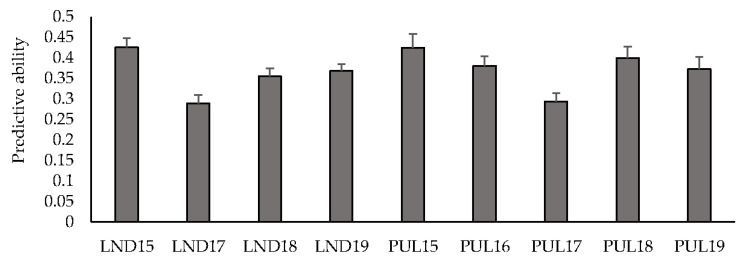
Predictive ability under cross-validations for grain yield across nine environments using an IBCF recommender system. Bars indicate standard errors.

Mean predictive ability across multiple environments for GY using an IBCF recommender approach ranged between 0.29 (LND17) and 0.43 (LND15) (Figure 1). Predicting within the LND environments resulted in a mean predictive ability of 0.36, whereas predicting the PUL environments resulted in an average predictive ability of 0.37. Predicting HD resulted in a mean predictive ability of 0.59, where PUL17 had the highest mean predictive ability (0.90), followed by PUL16 (0.86), and PUL18 (0.82) (Table S5). PH had a mean predictive ability of 0.55, where PUL17 had the highest mean predictive ability (0.82). Predicting the LND environments resulted in a mean of 0.44, whereas predicting within the PUL environments resulted in an average predictive ability of 0.64.

### 3.3. Predictive Ability for Grain Yield Using Secondary Correlated Traits

**Figure 2:**
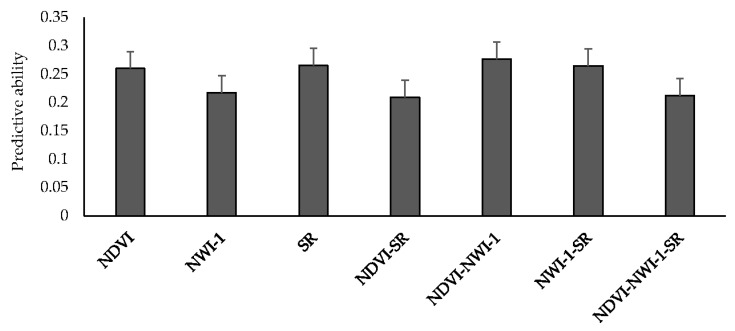
Mean predictive ability for grain yield across different environments using spectral indices collected from high-throughput field phenotyping under an IBCF (item-based collaborative filtering) recommender system. NDVI—Normalized Difference Vegetative Index; NWI-1—Normalized Water Index-1; SR—Simple Ratio. Bars indicate standard errors.

Mean predictive ability for GY using spectral indices from high-throughput field phenotyping ranged between 0.21 (NDVI-SR and NDVI-NWI-1-SR) and 0.28 (NDVI-NWI-1) (Figure 2). Altogether, there were no significant differences when different spectral indices were used to predict yield. Using one and two indices resulted in a 19% improvement in predictive ability compared to using all three indices (0.25 vs. 0.21). Predicting GY for LND17 using SRI resulted in the highest predictive ability (0.51), followed by LND15 (0.26), and PUL15 (0.22). Across environments, predicting yield using NDVI, NWI-1, and SR resulted in predictive ability values of 0.26, 0.22, and 0.27, respectively. Average correlation values between spectral traits and GY across environments were 0.12 (NDVI), −0.13 (NWI-1), and 0.11 (SR) (Table S6). Among the environments, LND17 had the highest average correlation for spectral traits and GY (0.19), followed by LND15 (0.08), and LND18 and PUL15 (0.05).

### 3.4. Predictive Ability Using Genomic Marker Information

**Figure 3:**
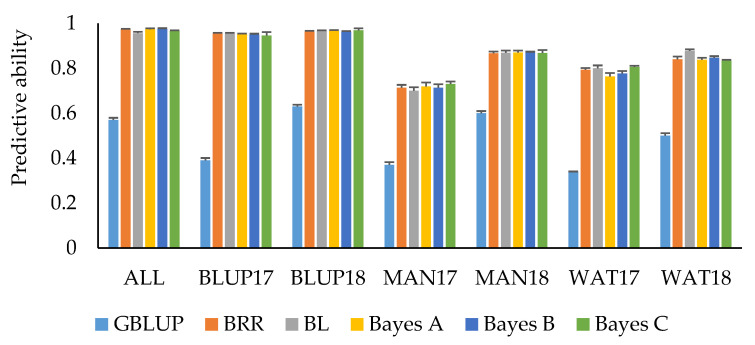
Mean predictive ability for snow mold tolerance across different environments and models. Trait values were adjusted using Bayesian models for IBCF. ALL—BLUP across all environments; BLUP17—BLUP across 2017 growing season; BLUP18—BLUP across 2018 growing season; MAN17—BLUE for Mansfield, WA, for 2017; MAN18—BLUE for Mansfield, WA, for 2018; WAT17—BLUE for Waterville, WA, for 2017; WAT18—BLUE for Waterville, WA, for 2018. BRR—Bayesian Ridge Regression; BL—Bayesian LASSO. GBLUP is a univariate, non-IBCF genomic BLUP model. Bars indicate standard errors.

Predictions by trait adjustments using SNP marker information through Bayesian regression models and implementing an IBCF recommender approach resulted in an overall improvement of predictive ability compared to the other GS scenarios (Table 5). PH had the highest mean predictive ability (0.57), followed by HD (0.55), and GY (0.51). Bayesian LASSO consistently showed the highest mean predictive ability for these traits compared to the other Bayesian models, although no significant differences for predictive ability among the models were observed. For GY, using Bayesian LASSO resulted in a mean predictive ability of 0.54, whereas using Bayes A and Bayes C for trait adjustments resulted in a predictive ability of 0.52 and 0.51, respectively. Using Bayesian Ridge Regression and Bayes B models resulted in a mean predictive ability of 0.50. Using Bayesian LASSO and Bayesian Ridge Regression for predicting HD resulted in a predictive ability of 0.57, whereas Bayes A, Bayes B, and Bayes C have comparable mean predictive ability (0.53–0.54). For PH, using Bayesian LASSO resulted in a mean predictive ability of 0.58, Bayesian Ridge Regression, Bayes A, and Bayes B in a predictive ability of 0.57, and Bayes C in a predictive ability of 0.56. Predictive ability for SMT was moderate to high across datasets. Predicting SMT resulted in a mean predictive ability of 0.87 for Bayesian Ridge Regression, Bayes A, B, and C, whereas for Bayesian LASSO a mean predictive ability of 0.88 was observed (Figure 3). Cross-validations for the ALL, BLUP17, and BLUP18 datasets resulted in predictive ability greater than 0.90 across different Bayesian models for SMT. Overall, predictive ability resulting from trait adjustments using Bayesian models for IBCF across the four traits was 81% higher compared to using GBLUP model (Table 6; Figure 3). The time it took to execute the IBCF algorithm with phenotypic trait adjustments for 15,229 SNP markers was ~64 min for three traits, nine environments, and 4104 observations using an Intel(R) Core(TM) i5-7200U CPU @ 2.50GHz, 2701 Mhz logical processor.

## 4. Discussion

The potential of a multi-trait, multi-environment IBCF recommender system for GS was assessed across different traits for winter wheat lines evaluated in US Pacific Northwest growing conditions. In the current study, we demonstrated the successful implementation of this approach in the context of a winter wheat breeding program, particularly in using genomic marker information for trait adjustments and improving the predictive ability of GS for yield, agronomic, and disease resistance traits.

Predicting a trait for a single year using combined information from multiple years would be the most effective strategy when predicting across years using the IBCF approach. It was observed that the correlation between years played an important role in achieving optimal cross-validation accuracies. Predicting performance of single years for GY in LND (average correlation *r* between environments of 0.21) resulted in higher mean predictive ability compared to PUL (mean *r* of 0.08) (0.16 vs. 0.03). The correlation between environments could therefore be regarded as the major driver for increased predictive ability across years. Likewise, PH, which had higher mean correlations between environments (*r* = 0.11), had the highest predictive ability overall (0.29) compared to GY and HD. These observations were consistent with studies [13,14,15] which previously demonstrated that for predicting complex traits, an IBCF approach would be most efficient if the correlation between environments were moderate to high. A lack of correlation between years would therefore be unfavorable for implementing a recommender approach for GS as low or negative correlations between environments could result in low predictive ability [2]. Further, the presence of genotype-by-environment (G x E) interactions, which could result in ‘weak’ correlations among years, especially when one year is different from the others, could impose challenges and limitations in the implementation of recommender systems for GS in breeding programs [13]. The variability in terms of weather patterns, for example, could have resulted in the differences across the environments. The 2015 growing season was considered to be having one of the warmest Spring temperatures in PUL at 22 °C (72 °F) (Loyd and Hoogenboom [29] https://weather.wsu.edu/index.php?page=AWN_Spring_2015_Weather_Review; retrieved 7 May 2020), whereas the 2018 season one of the wettest, with >10 consecutive days with precipitation in both LND and PUL environments (Washington State University AgWeatherNet; http://weather.wsu.edu/; retrieved 7 May 2020; [30]). Selecting stable lines with minimal G x E effects to ensure better correlation of phenotypes across years would therefore be crucial in the success of an IBCF recommender system for predicting future performance of lines in breeding programs. Recently, analyses of G x E interactions for this population of US Pacific Northwest winter wheat lines was conducted and stable lines based on AMMI and Finlay-Wilkinson regression coefficients were identified [20]. Furthermore, genetic mapping for yield and yield stability identified loci controlling both sets of traits demonstrating the potential of simultaneously selecting stable and higher-yielding lines [20].

Using secondary correlated traits that are more heritable than the target trait for GS has been shown to improve predictive ability [17,19,31,32]. Predicting yield using spectral traits collected from high-throughput phenotyping using the IBCF recommender system resulted in a 50% higher predictive ability compared with using single and multi-trait partial least square regression models [17] (0.24 vs. 0.16). Our results were comparable to Juliana et al. [15] who observed an average predictive ability of 0.22 in spring wheat lines with a narrow range of heading dates when green NVDI was used to predict GY in an IBCF approach. In the current study, using two traits instead of three resulted in the highest predictive ability in which predicting GY using NDVI-NWI-1 and NWI-1-SR resulted in a mean predictive ability of 0.28 and 0.26, respectively. Collinearity which could affect model predictive ability could result from including too many traits as predictors [33]; hence, using only a few highly heritable traits with high correlation with the target trait might be the ideal strategy to achieve better predictive ability in this GS scenario.

An increased correlation between GY and spectral indices was also related to improved predictive ability, similar in the case of predicting across years. Including spectral traits as fixed effects in ridge regression prediction models, nevertheless, might still be more advantageous in improving predictive ability compared to using a recommender approach for GS. Previously, a mean predictive ability of 0.43 for GY was observed using the same population of Pacific Northwest winter wheat lines when NDVI, NWI-1, and SR were included as fixed effects in an RRBLUP prediction model [19]. Canopy temperature and NDVI resulted in a 70% increase in predictive ability when predicting GY using multivariate models [34]. In other studies, incorporating markers for major growth and developmental genes such as *Vrn* and *Ppd* as fixed effects in the model improved predictive ability for GY, where combining multiple genes in the model resulted in optimal values [35,36]. Using GWAS-derived markers as fixed effects was also observed to have positive effects on the predictive ability for yield, stability, and related traits [37,38]. Predictive ability for GY using secondary spectral traits under an IBCF approach is ultimately a factor of the strength of correlation between traits across different environments.

Incorporating genomic information from molecular marker data under the IBCF recommender approach improved predictive ability for yield and agronomic traits compared to the other GS scenarios evaluated in the current study. By default, the IBCF algorithm does not allow for the direct incorporation of genomic data and therefore it is necessary to perform trait adjustments by markers using regression models first to obtain breeding values. Although we observed no significant differences among the various Bayesian regression models used for trait adjustments, using Bayesian LASSO resulted in the highest mean predictive ability across the datasets, indicating that this model could be preferentially used to achieve optimal predictive ability under an IBCF approach. Negative prediction accuracies were nevertheless still observed for the LND18 and PUL15 datasets, even when traits were already adjusted for marker effects. One disadvantage of IBCF is that poor predictions could result when genomic relationship matrices that do not represent the phenotypic correlations between genotypes are used as proxy values in performing predictions [14]. This could be the reason why negative prediction accuracies were observed for these datasets. In the present study, using an Illumina SNP chip for genotyping lines resulted in a moderate to high predictive ability across different environments for the evaluated traits. In the case of using genotyping-by-sequencing (GBS) markers, where there is an abundance of missing data for GS, imputation of missing markers can be carried out when there is a high correlation between the column of SNPs [14], consequently allowing for the effective implementation of an IBCF approach using GBS-derived SNP markers.

Among the advantages of an IBCF recommender approach in the context of GS is that its implementation is fast, straightforward, and does not require a lot of computing power [14,16]. However, in the current study, one drawback observed for the IBCF approach is its execution time. Compared to a univariate GBLUP model implemented in iPat, the time to execute an IBCF analysis where trait values were adjusted for marker effects using Bayesian models was longer (mean of ~64 min vs. ~13 min. for nine environments, 456 lines, and 15,229 SNP markers across three traits). Using a recommender system for moderate to large datasets would nonetheless still be a desirable alternative to the traditional GS models used in the breeding program in terms of achieving competitive prediction accuracies. When compared to other recommender approach such as matrix factorization, IBCF was also previously observed to be a better method for predicting GY, PH, and HD in wheat [14]. Finally, the IBCF.MTME is not a model-based approach, and hence it cannot estimate genetic variances or variance components [16], but nonetheless could still be used as an alternative approach for predicting traits for GS in plant breeding programs. In the future, the predictive power of other approaches for phenotypic trait adjustments such as generalized linear models via penalized maximum likelihood and frequentist LASSO (‘glmnet’ package in R; [39,40]) and non-local priors [41,42], and multivariate GBLUP [43] and compound symmetry GBLUP models should also be evaluated and compared with IBCF recommender system in the context of multi-environment, multi-trait GS. Exploring alternative strategies could facilitate the improvement of predictive ability for recommender approaches and other prediction models for GS in plant breeding programs [16].

## 5. Conclusions

The potential of using an IBCF recommender approach for GS was demonstrated for empirical datasets for GY, PH, and HD in a winter wheat breeding program. Low accuracies were observed for those datasets having low correlations, hence correlations among environments were crucial in achieving optimal prediction accuracies across years or growing seasons. Combining multiple secondary and highly heritable correlated spectral traits collected from high-throughput phenotyping would be ideal to improve predictive ability for GY. Trait adjustments through incorporating genomic marker information using Bayesian linear regression models resulted in the highest predictive ability among the GS scenarios for a recommender approach. Moreover, the predictive ability of predictions using IBCF was higher compared to using a GBLUP model, although the execution time for IBCF was slower. Altogether, our results indicate that using a recommender system such as IBCF as an alternative model should be considered in the context of multi-trait, multi-environment trials for GS in wheat breeding programs.

## Figures and Tables

**Table 1:** Sample phenotypic data for five lines and four trait–environment combinations (A, B, C, and D).

Line	A	B	C	D
1	x_A1_	2.00	1.50	2.25
2	1.75	x_B2_	2.25	3.00
3	1.50	2.25	x_C3_	2.75
4	2.00	3.00	2.00	x_D4_
5	1.25	2.75	2.00	3.00
Mean	1.63	2.50	1.94	2.75
Std. dev	0.28	0.40	0.27	0.31

**Table 2:** Adjusted (standardized) phenotypic data from Table 1.

Line	A	B	C	D
1	x_A1_	−1.27	−1.61	−1.63
2	0.45	x_B2_	1.15	0.82
3	−0.45	−0.63	x_C3_	0.0
4	1.34	1.27	0.23	x_D4_
5	−1.34	0.63	0.23	0.82

**Table 3:** Correlation between each trait–environment combination.

	A	B	C	D
**A**	1.00	0.14	−0.31	−0.55
**B**	0.14	1.00	0.44	0.52
**C**	−0.31	0.435	1.00	0.94
**D**	−0.55	0.516	0.94	1.00

**Table 4:** Predictive ability for grain yield by training different years to predict performance in subsequent growing seasons across environments using an IBCF (item-based collaborative filtering) approach.

Location	Training Year(s)	Test Year	Predictive Ability	MAAPE
LND	2015	2017	−0.03	0.22
LND	2015	2018	−0.18	0.45
LND	2015	2019	0.43	0.28
LND	2015, 2017	2018	−0.18	0.39
LND	2015, 2017	2019	0.43	0.21
LND	2015, 2017, 2018	2019	0.46	0.16
PUL	2015	2016	−0.20	0.31
PUL	2015	2017	−0.08	0.24
PUL	2015	2018	−0.21	0.24
PUL	2015	2019	0.43	0.25
PUL	2015, 2016	2017	−0.12	0.27
PUL	2015, 2016	2018	−0.21	0.33
PUL	2015, 2016	2019	0.36	0.29
PUL	2015, 2016, 2017	2018	−0.16	0.35
PUL	2015, 2016, 2017	2019	0.19	0.21
PUL	2015, 2016, 2017, 2018	2019	0.32	0.14

MAAPE—mean arctangent percentage error.

**Table 5:** Predictive ability from cross-validations for grain yield, heading date, and plant height for phenotypic values adjusted using genomic (marker) information from various Bayesian models.

Model	Env	Grain Yield		Heading Date		Plant Height	
		Pred. Ability (PA)	SE_PA ^2^	Pred. Ability (PA)	SE_PA	Pred. Ability (PA)	SE_PA
BRR ^1^	LND15	0.20	0.03	0.62	6.10 × 10^−3^	0.61	0.02
	LND17	0.66	0.02	0.36	0.03	0.36	0.03
	LND18	0.60	0.02	−0.15	0.03	0.56	0.02
	LND19	0.52	0.02	0.57	0.02	0.27	0.02
	PUL15	0.49	0.03	0.18	0.04	−0.18	0.03
	PUL16	0.41	0.01	0.85	0.02	0.86	7.50 × 10^−3^
	PUL17	0.56	0.02	0.93	5.90 × 10^−3^	0.92	7.10 × 10^−3^
	PUL18	0.47	0.03	0.88	0.01	0.86	0.01
	PUL19	0.58	0.02	0.90	6.00 × 10^−3^	0.89	6.20 × 10^−3^
Bayes A	LND15	0.19	0.04	0.58	0.01	0.61	0.03
	LND17	0.69	0.01	0.24	0.03	0.37	0.02
	LND18	0.58	0.03	−0.24	0.03	0.56	0.02
	LND19	0.59	0.02	0.57	0.02	0.22	0.02
	PUL15	0.55	0.02	0.17	0.02	−0.12	0.03
	PUL16	0.42	0.03	0.83	0.02	0.83	7.80
	PUL17	0.60	0.03	0.94	3.80 × 10^−3^	0.91	4.30
	PUL18	0.45	0.03	0.86	6.80 × 10^−3^	0.87	7.10
	PUL19	0.61	0.01	0.90	5.10 × 10^−3^	0.88	5.90
Bayes B	LND15	0.25	0.03	0.65	0.01	0.63	0.02
	LND17	0.62	0.02	0.30	0.02	0.33	0.03
	LND18	0.55	0.02	−0.17	0.03	0.59	0.01
	LND19	0.55	0.03	0.52	0.01	0.23	0.02
	PUL15	0.48	0.03	0.15	0.04	−0.14	0.02
	PUL16	0.41	0.03	0.83	0.02	0.83	0.01
	PUL17	0.57	0.02	0.93	5.00 × 10^−3^	0.92	4.50 × 10^−3^
	PUL18	0.45	0.02	0.87	6.20 × 10^−3^	0.87	0.01
	PUL19	0.58	0.02	0.90	5.00 × 10^−3^	0.87	0.01
Bayes C	LND15	0.28	0.03	0.64	0.02	0.56	0.02
	LND17	0.69	0.02	0.24	0.03	0.36	0.03
	LND18	0.55	0.02	−0.22	0.03	0.55	0.03
	LND19	0.51	0.02	0.54	0.02	0.31	0.03
	PUL15	0.52	0.013	0.09	0.03	−0.19	0.02
	PUL16	0.40	0.03	0.86	0.01	0.84	8.50 × 10^−3^
	PUL17	0.60	0.02	0.93	5.00 × 10^−3^	0.91	4.70 × 10^−3^
	PUL18	0.44	0.01	0.87	9.20 × 10^−3^	0.86	6.10 × 10^−3^
	PUL19	0.56	0.01	0.90	4.90 × 10^−3^	0.88	6.30 × 10^−3^
BL ^3^	LND15	0.34	0.03	0.64	0.02	0.66	0.02
	LND17	0.66	0.01	0.30	0.04	0.40	0.04
	LND18	0.61	0.02	−0.22	0.03	0.59	0.02
	LND19	0.56	0.02	0.59	0.02	0.25	0.03
	PUL15	0.57	0.02	0.24	0.03	−0.20	0.03
	PUL16	0.42	0.03	0.88	9.70 × 10^−3^	0.86	7.90 × 10^−3^
	PUL17	0.61	0.02	0.93	5.20 × 10^−3^	0.92	4.40 × 10^−3^
	PUL18	0.43	0.01	0.86	6.80 × 10^−3^	0.86	0.01
	PUL19	0.65	0.03	0.91	6.60 × 10^−3^	0.90	6.70 × 10^−3^

^1^ Bayesian Ridge Regression. ^2^ Standard error of predictive ability. ^3^ Bayesian LASSO.

**Table 6:** Predictive ability for grain yield, heading date, and plant height using a genomic BLUP (GBLUP) model across different environments.

Env	Grain Yield		Heading Date		Plant Height	
	Pred. Ability	RMSE	Pred. Ability	RMSE	Pred. Ability	RMSE
LND15	0.47	0.47	0.59	2.13	0.63	5.91
LND17	0.39	0.68	0.30	2.23	0.27	8.19
LND18	0.44	0.86	0.25	2.30	0.29	7.08
LND19	0.39	0.56	0.68	1.82	0.38	6.03
PUL15	0.41	0.85	0.20	2.20	0.18	6.86
PUL16	0.43	0.78	0.71	1.54	0.42	6.90
PUL17	0.39	0.63	0.69	2.26	0.51	2.55
PUL18	0.42	0.79	0.64	1.60	0.52	6.95
PUL19	0.36	1.14	0.61	2.23	0.58	6.32

RMSE—root mean square error.

## Supplementary Materials

The following are available online at https://www.mdpi.com/2073-4425/11/7/779/s1, Table S1: Predictive ability for heading date and plant height by training different years to predict performance in subsequent growing seasons across environments using an IBCF (item-based collaborative filtering) approach, Table S2: Pairwise correlation between environments for grain yield, Table S3: Pairwise correlations between environments for heading date, Table S4: Pairwise correlations between environments for plant height, Table S5: Predictive ability under cross-validations for heading date and plant height across nine environments using an IBCF (item-based collaborative filtering) recommender system, Table S6: Correlation between spectral traits and grain yield across different environments.
