# Supplementary material for: Genomic Selection in Winter Wheat Breeding Using a Recommender Approach
Source: Genes (Basel). 2020 Jul 11;11(7):779. doi: 10.3390/genes11070779 (PMC7397162; doi:10.3390/genes11070779)
Supplement: Supplementary file 1 [file genes-11-00779-s001.pdf]

Table S1. Predictive ability for heading date and plant height by training different years to predict performance in subsequent growing seasons across environments using an IBCF (item-based collaborative filtering) approach.

Test Trait	Location	Training year(s)	Testing year	Predictive ability	MAAPE
Heading date	PUL	2015, 2016	2018	0.07	0.04
Heading date	PUL	2015, 2016, 2017	2019	0.07	0.02
Heading date	PUL	2015, 2016, 2017, 2018	2019	0.07	0.03
Heading date	PUL	2015	2018	0.06	0.01
Heading date	PUL	2015	2016	0.05	0.04
Heading date	PUL	2015	2019	0.05	0.07
Heading date	PUL	2015, 2016	2019	0.05	0.05
Heading date	LND	2015	2019	-0.05	0.04
Heading date	LND	2015, 2017	2019	-0.08	0.02
Heading date	LND	2015, 2017, 2018	2019	-0.08	0.02
Heading date	PUL	2015, 2016, 2017	2018	-0.08	0.08
Heading date	PUL	2015	2017	-0.09	0.10
Heading date	PUL	2015, 2016	2017	-0.09	0.09
Heading date	LND	2015	2018	-0.12	0.16
Heading date	LND	2015, 2017	2018	-0.13	0.07
Heading date	LND	2015	2017	-0.14	0.07
Plant height	LND	2015, 2017, 2018	2019	0.41	0.07
Plant height	PUL	2015, 2016, 2017, 2018	2019	0.40	0.28
Plant height	PUL	2015	2019	0.39	0.20
Plant height	PUL	2015, 2016, 2017	2019	0.36	0.18
Plant height	PUL	2015, 2016	2019	0.33	0.20
Plant height	LND	2015	2019	0.31	0.11
Plant height	LND	2015, 2017	2019	0.28	0.07
Plant height	PUL	2015, 2016	2017	0.06	0.97
Plant height	PUL	2015	2017	0.05	0.96
Plant height	PUL	2015, 2016	2018	-0.05	0.15
Plant height	PUL	2015, 2016, 2017	2018	-0.09	0.26
Plant height	LND	2015	2017	-0.11	0.13
Plant height	PUL	2015	2018	-0.12	0.13
Plant height	LND	2015	2018	-0.18	0.18
Plant height	LND	2015, 2017	2018	-0.18	0.12
Plant height	PUL	2015	2016	-0.23	0.08

MAAPE- Mean arctangent percentage error

Table S2. Pairwise genetic correlation between environments for grain yield.

	LND15	LND17	LND18	LND19	PUL15	PUL16	PUL17	PUL18
**LND17**	0.26***	-						
**LND18**	0.14*	0.28***	-					
**LND19**	0.05*	0.25***	0.26***	-				
**PUL15**	0.32***	0.14*	0.02	0.08	-			
**PUL16**	0.03	0.01	0.09	0.05	0.05	-		
**PUL17**	-0.02	-0.08	-0.04	0.02	0.24***	0.13*	-	
**PUL18**	-0.13*	0.02	0.014	0.01	0.21***	0.14*	0.23***	-
**PUL19**	0.12*	-0.05	-0.07	-0.09*	0.29***	0.18**	0.20***	0.17**

*** - Significant at $P < 0.0001$

**- Significant at $P < 0.001$

*- Significant at $P < 0.05$

Table S3. Pairwise genetic correlation between environments for heading date.

	LND15	LND17	LND18	LND19	PUL15	PUL16	PUL17	PUL18
**LND17**	0.02	-						
**LND18**	-0.14*	0.39***	-					
**LND19**	0.59***	0.02	-0.19	-				
**PUL15**	-0.01	-0.15*	-0.17**	0.04	-			
**PUL16**	0.48***	-0.01	-0.09	0.72***	0.04	-		
**PUL17**	0.47***	0.03	-0.004	0.76***	-0.006	0.8***	-	
**PUL18**	0.42***	0.04	0.02	0.68***	0.03	0.71***	0.84***	-
**PUL19**	0.48***	0.01	-0.04	0.71***	0.02	0.75***	0.86***	0.74***

*** - Significant at $P < 0.0001$

**- Significant at $P < 0.001$

*- Significant at $P < 0.05$

Table S4. Pairwise genetic correlation between environments for plant height.

	LND15	LND17	LND18	LND19	PUL15	PUL16	PUL17	PUL18
**LND17**	0.06	-						
**LND18**	0.15*	0.33	-					
**LND19**	0.31***	0.05	0.07	-				
**PUL15**	0.01	-0.01	0.05	0.11	-			
**PUL16**	0.5***	0.12*	0.14*	0.44***	0.01	-		
**PUL17**	0.52***	0.07	0.19***	0.47***	0.02	0.75***	-	
**PUL18**	0.45***	-0.02	0.14*	0.46***	0.02	0.68***	0.82***	-
**PUL19**	0.48***	0.12*	0.22***	0.43***	-0.01	0.69***	0.78***	0.72***

*** - Significant at $P < 0.0001$

**- Significant at $P < 0.001$

*- Significant at $P < 0.05$

Table S5. Predictive ability under cross-validations for heading date and plant height across nine environments using an IBCF (item-based collaborative filtering) recommender system.

Trait	Env.	Predictive ability (PA)	PA_SE
Heading date	LND15	0.57	0.021
Heading date	LND17	0.20	0.027
Heading date	LND18	0.31	0.0237
Heading date	LND19	0.78	0.0112
Heading date	PUL15	0.03	0.0367
Heading date	PUL16	0.86	0.007
Heading date	PUL17	0.90	0.0041
Heading date	PUL18	0.82	0.0071
Heading date	PUL19	0.82	0.0109
Plant height	LND15	0.56	0.0082
Plant height	LND17	0.30	0.027
Plant height	LND18	0.41	0.026
Plant height	LND19	0.48	0.0138
Plant height	PUL15	0.05	0.03
Plant height	PUL16	0.77	0.0106
Plant height	PUL17	0.82	0.0119
Plant height	PUL18	0.80	0.0139
Plant height	PUL19	0.77	0.0096

Table S6. Genetic correlation between spectral traits and grain yield across different environments.

Spectral trait	LND15	LND17	LND18	PUL15	PUL16	PUL17	PUL18
NDVI	0.27***	0.58***	0.13*	0.13*	-0.06	-0.09	-0.11*
NWI-1	-0.23***	-0.59***	-0.11*	-0.13*	0.02	0.10	0.06
SR	0.19***	0.58***	0.09	0.11*	-0.07	-0.08	-0.06

*** - Significant at $P < 0.0001$

**- Significant at $P < 0.001$

*- Significant at $P < 0.05$
